# Pore pressure evolution and mass loss of broken gangue during the seepage

**DOI:** 10.1098/rsos.180307

**Published:** 2018-10-10

**Authors:** Jixiong Zhang, Yu Liu, Nan Zhou, Meng Li

**Affiliations:** 1 State Key Laboratory of Coal Resources and Safety Mining, China University of Mining and Technology, Beijing, People's Republic of China; 2 School of Mechanic Engineering, Jiangsu Normal University, Xuzhou, People's Republic of China

**Keywords:** crushed gangue, permeability, pressure evolution, mass loss

## Abstract

Broken gangue consists of different particles, and it has more complicated seepage characteristics than intact rock sample. Using the self-designed instrument, the permeability, mass loss and pore pressure of crushed gangue during the seepage are tested. The result shows that permeability parameter *k* of crushed rock has a polynomial relationship with effective stress *σ*′ in inverse proportion, and permeability parameter *β* of crushed gangue has power exponent relationship with effective stress *σ*′ increasing in direct proportion. The particle size of 8.0–10.0 mm has a good support effect. The inner pressure of crushed rock is mostly linear distribution along the tube wall. After the seepage, mass loss of broken gangue mainly increases with large particle size out of proportion.

## 1. Introduction

**Figure 1.**
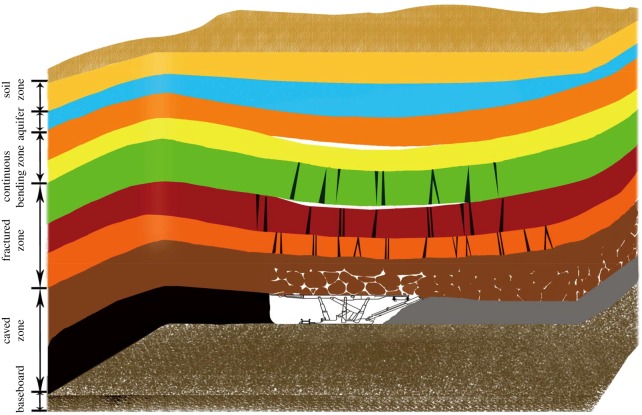
Three zones of coal mine during longwall mining.

Three zones of disturbances, caving zone, fissure zone and slow subsidence zone, may occur during longwall top coal caving, as shown in figure 1. The consolidation of the caved zone after mining operations will be re-compacted to new equilibrium [1,2], which is closely related to gas-drainage, mine safety and water conservation [3–5]. The compaction and permeability of broken rocks are coupled and need to be discussed (table 1).

**Table 1.** Nomenclature.

list of symbols	
*d*	diameter of broken gangue
*h*_0_	initial height of the broken gangue sample
*H*	height of the broken gangue sample
*H*_1_	cylinder tube height (*H*_1_)
*H*_2_	measurement dimension between cylinder tube and overflow cover
*H*_3_	thickness of the overflow cover
*H*_4_	thickness of overflow cylinder
*H*_5_	height of higher porous board
*H*_6_	thickness of porous board
*k*	permeability (L^2^)
*L*	sample length (L)
*m*	mass of the broken gangue sample (M)
*p*	pore pressure (ML^−1^ T^−2^)
*p_a_*	pore pressure at the intake boundary (ML^−1^ T^−2^)
*p_b_*	pore pressure connected with atmosphere (ML^−1^ T^−2^)
*p*_1_	pressure of pore 1 (ML^−1^ T^−2^)
*p*_2_	pressure of pore 2 (ML^−1^ T^−2^)
*p*_3_	pressure of pore 3 (ML^−1^ T^−2^)
*p*_4_	pressure of pore 4 (ML^−1^ T^−2^)
*p*_5_	pressure of pore 5 (ML^−1^ T^−2^)
*Q*	cross-section area of the cylinder tube (L^2^)
Re	Reynold number (–)
*S*	axial displacement (L)
*V*	water flow velocity (LT^−1^)
*x*	vertical axis along the centre of the specimen
*∂*	partial differential operator (−)
*∂*()/*∂x*	Nabla operator (L^−1^)
*β*	non-Darcy coefficient (L^−1^)
*ρ_s_*	mass density (ML^−3^)
*ρ*_w_	water density (ML^−3^)
*σ*	total stress (ML^−1^ T^−2^)
*σ^’^*	effective stress (ML^−1^ T^−2^)
*μ*	kinetic viscosity of water (ML^−1^ T^−1^)
*ϕ*	effective porosity of the broken gangue

Caved zone lies directly on the gob, re-compaction of caving zone will become the barrier for blocking water. In order to obtain the evolution of caved zone, therefore, it is important to take many factors into account, such as the compaction of broken rock [6], flow characteristics of broken rock [7] and the interaction of stress and deformation [8,9]. Many factors, such as force loading style [10], particle shape [11], shear strength [12] and fracture behaviour [13], may affect the compaction quality of broken rock and soils. During the compaction, the rock and soil may be crushed during the loading [14]; the permeability is influenced by many factors, such as the initial grading of the tested rock [15], the lithology [16], the geological framework [17], geometry and physical characteristics.

On the other hand, Darcy obtained the linear seepage through the permeability test of the homogeneous sand column in 1856. Investigations with experimental and numerical simulation about the permeability of broken rock and soil have been carried out, and substantial progress was made [18–22]. The relationship of porosity, effective stress and permeability was fitted into empirical formulae [23,24], and initial porosity, load histories and deformation properties were considered [25–27].

The inner compaction and pore pressure of broken rock during the loading is presented. According to the above works, the broken rock was thought as the integrated whole to discuss pressure gradient and seepage velocity, porosity evolution law under compression [28], fractal feature of broken rock under compression [29], and the relationship between permeability and porosity [30]. But the permeability and stress mechanism during the seepage in inner broken rock was not discussed under different loading of particle size. The set of broken rock seepage system was designed to study the inner pore pressure changing in broken rock. Based on the above analysis, this study further studied the seepage of broken gangue in different particle size by using hierarchical loading method to explore the internal penetration mechanism.

## 2. Experimental system and methods

### 2.1. Experiment instruction

**Figure 2.**
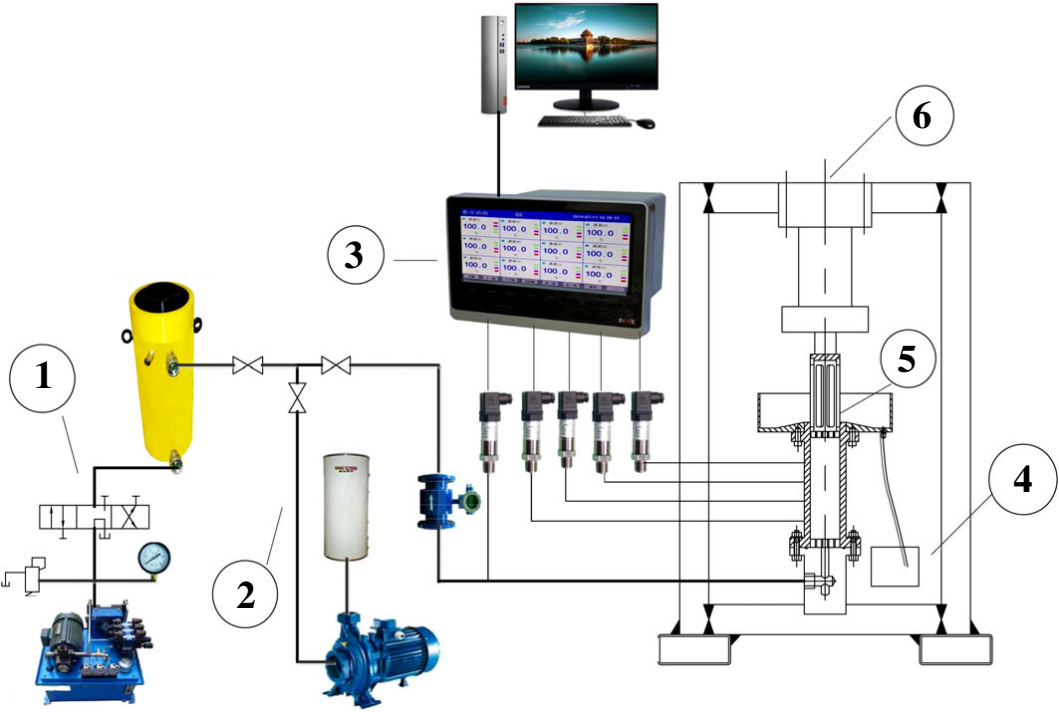
Test system of seepage. 1, water pressure supply system; 2, hydraulic pressurization equipment; 3, sewage collection equipment; 4, data acquisition equipment; 5, axial compression loading equipment; 6, self-made seepage test device.

The seepage system of crushed rock includes water pressure supply system (1), hydraulic pressurization equipment (2), sewage collection equipment (3), data acquisition equipment (4), axial compression loading equipment (5) and self-made seepage test device (6), as shown in figure 2.

The pore water supply equipment includes water pump, water pond, globe valve and double acting hydraulic cylinder, which can be injected into water for one part and oil for the other part. The hydraulic pressurization equipment consists of oil pump, selector valve, overflow valve and pressure gauge.

By adjusting selector valve and overflow valve, the hydraulic pressurization equipment can raise the water in the double acting hydraulic cylinder to several stable higher pressures.

**Figure 3.**
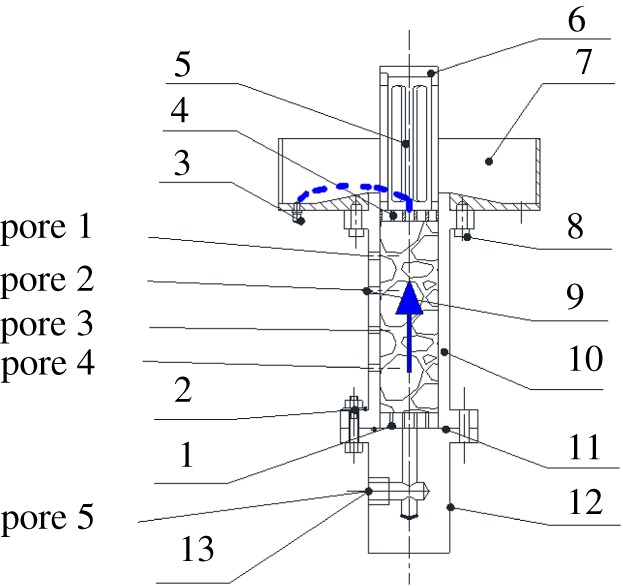
Seepage instrument. 1, lower porous board; 2, screw bolt; 3, outlet pipe; 4, higher porous board; 5, overflow cylinder; 6, overflow cover; 7, salver; 8, screw fastener; 9, pore (1,2,3,4,5); 10, cylinder tube; 11, lock ring; 12, bottom; 13, water inlet.

The self-made seepage test device consists of lower porous board (1), screw bolt (2), outlet pipe (3), higher porous board (4), overflow cylinder (5), overflow cover (6), salver (7), screw fastener (8), pore 1,2,3,4,5 (9), cylinder tube (10) lock ring (11), bottom (12), water inlet (13) as shown in figure 3. Pores 1,2,3,4 are at regular intervals along the cylinder tube (10).

As shown in figure 2, the seepage instrument was put in axial compression loading equipment; 10 mm diameter hole of the higher porous board (4 in figure 3) can allow little broken granular medium overflow.

### 2.2. Crushed gangue and specimen preparation

The gangue was taken from about −325 m strata, in Xiaojiawa coal mine in Shanxi province of People's Republic of China.

**Figure 4.**
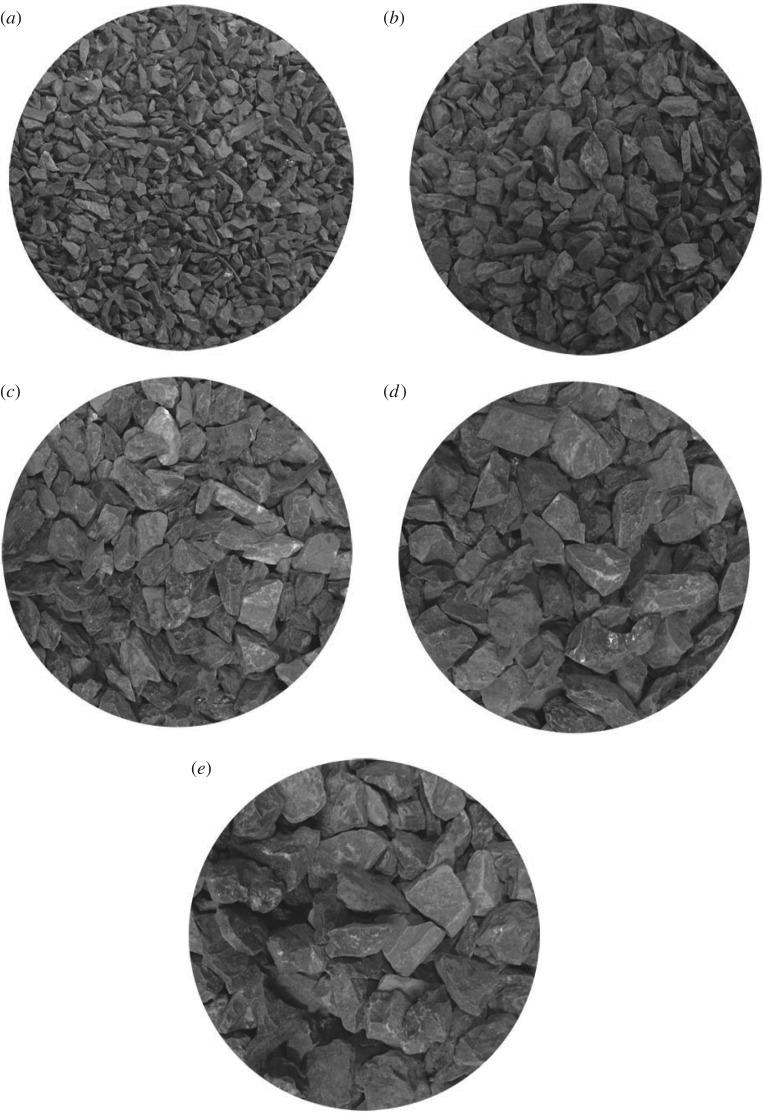
Crushed gangue (*a*) 2.5–5.0 mm, (*b*) 5.0–8.0 mm, (*c*) 8.0–10.0 mm, (*d*) 10.0–12.0 mm, (*e*) 12.0–15.0 mm.

The density of the gangue is *ρ_s_* = 2.32 × 10^3^ kg m^−3^, the crushed gangue is separated into five groups: a (2.5–5.0 mm), b (5.0–8.0 mm), c (8.0–10.0 mm), d (10.0–12.0 mm) and e (12.0–15.0 mm) screened by the griddle, as shown in figure 4.

### 2.3. Procedures

**Figure 5.**
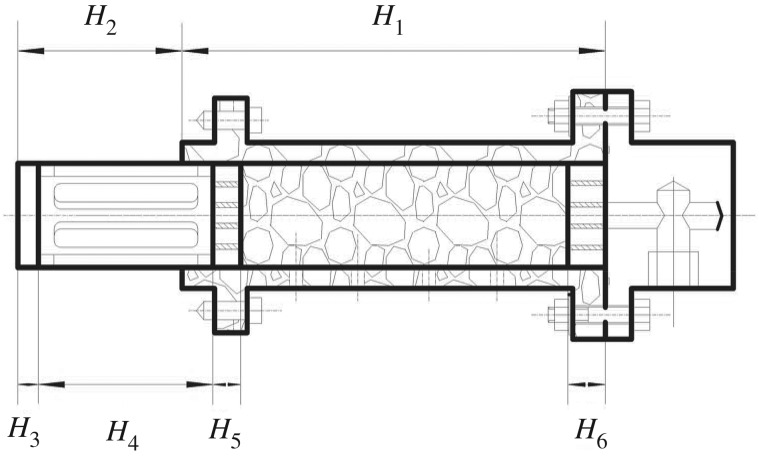
Sketch of calculating the height of the crushed gangue.

Crushed gangue samples are needed to be completely saturated by water before each test. The test steps are as follows: weigh 4500 g screened gangue, place the gangue into the cylinder tube, as shown in figure 5, where the cylinder tube height is *H*_1_, the measurement dimension between the cylinder tube and overflow cover *H*_2_ and the thickness of the overflow cover *H*_3_, the thickness of overflow cylinder *H*_4_, the height of higher porous board *H*_5_ and the height of lower porous board *H*_6_*.* Then the initial height *h_o_* = *H*_1_ + *H*_2_−*H*_3_−*H*_4_−*H*_5_−*H*_6_ can be calculated. Apply the axial load to the sample, and reach the force as the designed value, then keep it steady until the seepage process is finished.

**Figure 6.**
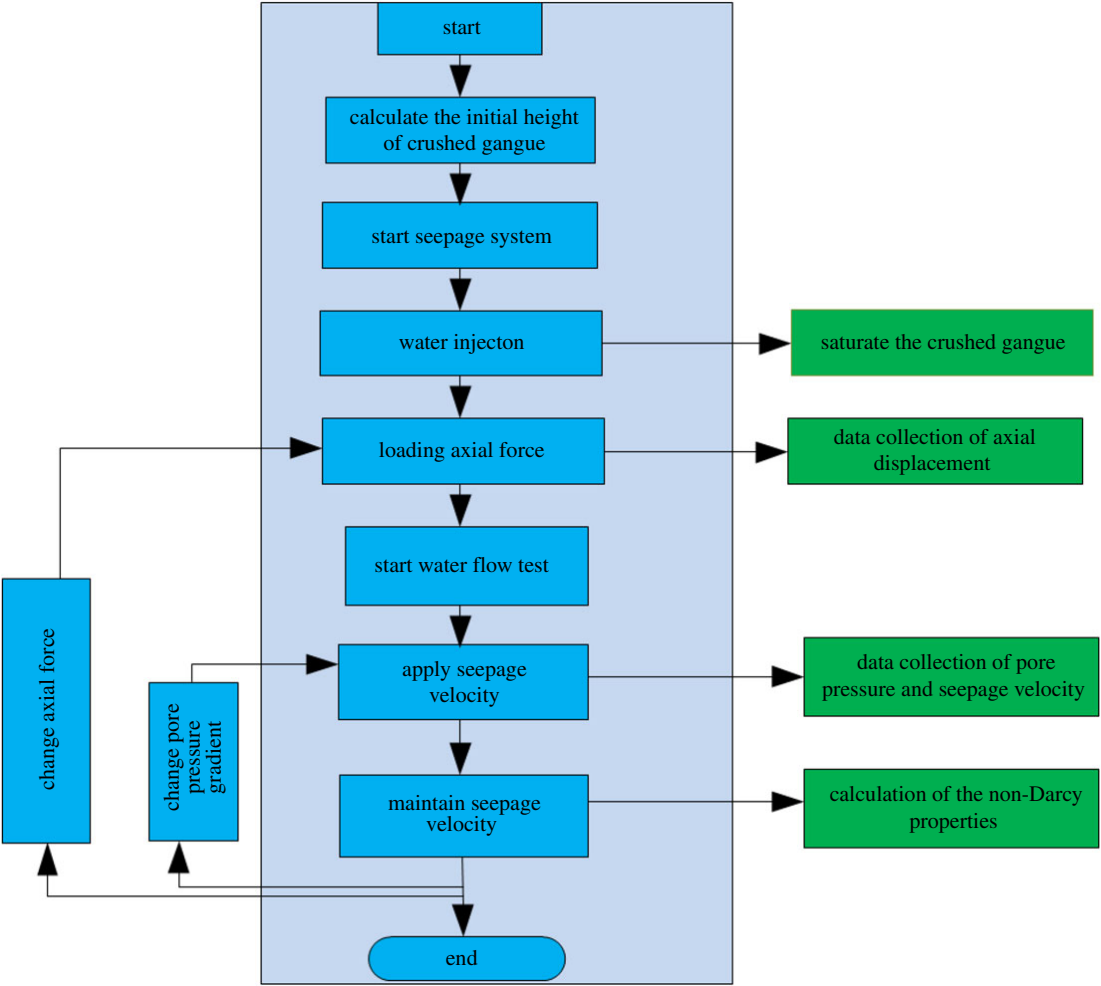
Flow chart of seepage.

The axial compression loading equipment exerts forces of 40, 60, 80, 100, 120 and 140 kN. The velocity of water can be controlled by the speed of hydro-cylinder's piston which can be pushed forward by the oil pump; the output pressure of oil pump can be controlled by a hydraulic valve, from 1, 2, 3 and 3.5 MPa. We can obtain the stable velocity after 60 s and the corresponding pressure gradient for each velocity. Figure 6 shows the flow chart of seepage.

### 2.4. Experimental methods

The relationship between pressure and flow velocity of water flow in the crushed rock can be expressed by Forchheimer formula [31]:

2.1 $$-\frac{\partial p}{\partial x}=\frac{\mu }{k}V+\rho_{w}\beta V^{2},$$

where **∂*p/*∂*x* is the pore pressure gradient, *μ* is the kinetic viscosity of the water, *k* is the permeability of the broken gangue, *V* is an average water flow velocity. *ρ*_w_ is the water density, *β* is the non-Darcy coefficient, *x* is the vertical axis along the centre of the specimen.

Through the test, the steady pore pressure-flow velocity *v* can be obtained. In our tests, the upstream end was connected with the atmosphere, in other words, the pressure of water in the outlet of broken gangue is zero.

There are four pores at regular intervals along the cylinder tube *H*_1_, which connected to four pressure sensors that could automatically record the pore water pressure *p* real-time. The downstream end of the rock specimen is connected to the pressure sensor which also automatically records the pore water pressure *p* over time *t*.

Then, the steady pore pressure gradient is calculated. If all the parameters on the right side of equation (2.2) do not change with *x*, then the pore pressure gradient **∂*p/*∂*x* is a constant. This can be determined by

2.2 $$\frac{\partial p}{\partial x}=-\frac{\;p_{a}-p_{b}}{L}=\frac{-p_{a1}}{L},$$

where *L* is the sample length, *p_a_* is the pore pressure at the intake boundary, *p_b_* is the pore pressure connected with the atmosphere. Then equation (2.3) is obtained as follows:

2.3 $$\frac{\;p_{a}}{L}=\frac{\mu }{k}V+\rho_{w}\beta V^{2}.$$

The permeability *k* and the non-Darcy coefficient are calculated by the relationship between the pressure gradient and fluid velocity curves (*p–V* curve).

For the crushed rocks, Reynolds number (Re) is defined as

2.4 Re=ρwVdμϕ,

where *d* is the average diameter of the grains, *ϕ* is an effective porosity of the broken gangue. The porosity *ϕ* of each displacement level can be developed as follows:

2.5 $$\phi =1-\frac{m}{\rho_{s}Q(h_{0}-S)},$$

where *m*, *ρ_s_* and *h*_0_ are the mass, mass density and the initial height of the broken gangue sample, respectively, *Q* is the cross-section area of the cylinder tube, and *S* is an axial displacement.

The testing fluid was water with density *ρ*_w_ *_=_* 1.0 × 10^3^ kg m^−3^ and kinetic viscosity *μ* = 1.01 × 10^−3^ Pa s at the standard state. We can calculate the Re according to equation (2.5) to prove the non-Darcy equation (equation (2.3)) can be used to model the water flow in crushed mudstones. The porosity *φ* of each displacement level was calculated based on equation (2.4), *φ*_min_ = 0.0401, *φ*_max_ = 0.2179. In the test, *V*_min_ = 8.49 × 10^−5^ m s^−1^, *V*_max_ = 5.30 × 10^−3^ m s^−1^. Putting *d* = 2.5–5.0 mm, *φ* = [0.0421, 0.1801], *ρ*_w_ = 1000 kg m^−3^ and *μ* = 1.01 × 10^−3^ Pa s into equation (2.5) gives *Re*_min_ = 2.496, *Re*_max_ = 36.42. This means non-Darcy flow can be observed in the test.

## 3. Results and discussion

### 3.1. Effective stress and permeability parameters

Terzaghi proposed the principle of effective stress in 1925 (equation (3.1)).

3.1 $$\sigma =\sigma^{\prime }+p,$$

where *σ* is the total stress, *σ*′ is an effective stress and *p* is the pore pressure.

**Figure 7.**
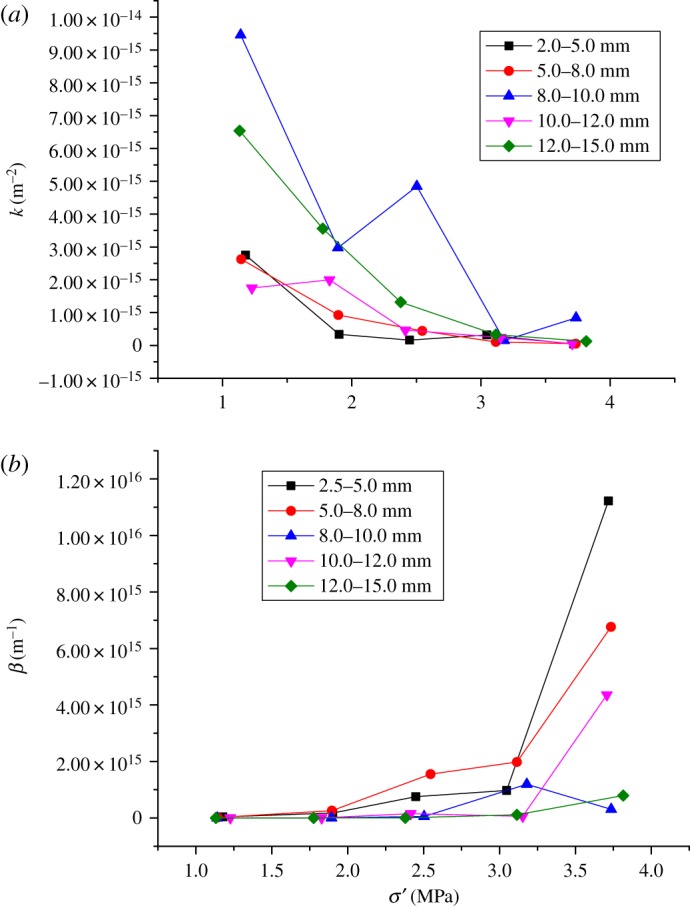
Relationship between effective stress and seepage parameters: (*a*) curves of effective stress and permeability and (*b*) curves of effective stress and non-Darcy factor.

In figure 7*a*, permeability parameter *k* decreases along with effective stress *σ*, and non-Darcy factor *β* increases with effective stress *σ*′ in figure 7*b*.

For the particle sizes of 8.0–10.0, 10.0–12.0, 12.0–15.0 mm, the permeability parameter *k* is significantly greater than that of the other two particle sizes of 2.5–5.0 and 5.0–8.0 mm under effective stress 3 MPa. The permeability parameter *k* of five particle sizes has a small gap with the continuous increase of effective stress. For 8.0–10.0 mm particle size, the tendency of the curve of appeared the sudden change at the effective stress 2.6 MPa. In other words, the permeability of that particle size is tortuous with the increase of the effective stress. The reason for that is when the effective stress increases, the change of particle size fracture and recombination is the largest, and the influence on permeability is also the largest.

When the effective stress is less than 2 MPa, the variation law of non-Darcy factor *β* of five particle sizes is similar. Non-Darcy factor *β* of 2.5–5.0 and 5.0–8.0 mm increases quickly when the effective stress exceeds 2.0 MPa.

Table 2 presents the fitting formulae which describe the relationship between effective stress and permeability parameter. Along with the increase of effective stress, the porosity will decrease, which results in the reduction of permeability.

**Table 2.** Fitting formulae between effective stress and permeability parameter. *σ*′, effective stress; *β*, non-Darcy factor, which reflects the permeability of crushed coal gangue from another perspective.

particle size (mm)	permeability *k* (m^−2^)	correlation coefficient	non-Darcy factor *β* (m^−1^)	correlation coefficient
2.5–5.0	*k* = 8.01 × 10^−16^ *σ*^2^ – 4.82 × 10^−15^ *σ*′ + 7.10 × 10^−15^	0.8930	*β* = 1.08 × 10^−29^ *σ*′^5.92^	0.9295
5.0–8.0	*k* = 5.36 × 10^−16^ *σ*′^2^ – 3.56 × 10^−15^ *σ*′ + 5.94 × 10^−15^	0.9884	*β* = 2.62 × 10^−34^ *σ*′^6.55^	0.9624
8.0–10.0	*k* = 1.20 × 10^−15^ *σ*^2^ – 9.01 × 10^−15^ *σ*′ + 1.77 × 10^−14^	0.8224	*β* = 7.37 × 10^−70^ *σ*′^11.14^	0.8943
10.0–12.0	*k* = 9.48 × 10^−17^ *σ*′^2^ – 1.28 × 10^−15^ *σ*′ + 3.42 × 10^−15^	0.8156	*β* = 4.16 × 10^−45^ *σ*′^7.84^	0.7219
12.0–15.0	*k* *=* *1.18* × 10^−15^ *σ*′*^2^*– *8.19* × 10^−15^ *σ*′ *+* *1.43* × 10^−14^	0.9984	*β* = 1.83 × 10^−62^ *σ*′^10.09^	0.8891

### 3.2. Compactness of broken gangue

**Figure 8.**
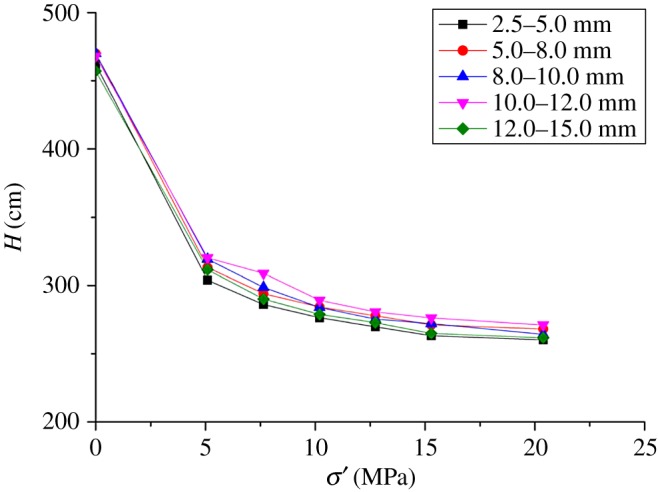
Deformation of broken gangue during the loading.

Table 3 and figure 8 show the height of broken gangue under different axial loading. For each particle size of broken gangue, record initial height and load the axial force 40, 60, 80, 100, 120 and 140 kN in proper order, and the different heights of different states are obtained.

**Table 3.** Height of broken gangue under different axial loading.

	height of broken gangue (mm)				
axial loading (kN)	2.5–5.0	5.0–8.0	8.0–10.0	10.0–12.0	12.0–15.0
0	462.10	470.23	470.81	468.64	457.75
40	303.85	313.26	319.29	320.31	311.57
60	285.99	293.99	298.63	308.97	290.06
80	276.29	284.28	283.90	289.15	278.81
100	269.73	277.73	275.36	280.59	272.75
120	263.15	271.15	272.08	276.14	264.85
140	260.15	268.15	264.19	271.10	261.65

During the deformation, the height of broken gangue does not show increases in proportion to the particle size of broken gangue. In table 3, 8.0–10.0 mm particle size has the highest initial height and the final one, which means the size of the rock has a good support effect. For the 2.5–5.0 mm particle size, the early deformation is large under loading 100 kN. With the loading force increasing, the trend of deformation is slowing down. For 12.0–15.0 mm, its condition is the opposite of 2.5–5.0 particle size under loading 100 kN. As the axial pressure continues to increase, compression trend of 12.0–15.0 mm is similar to that of other particle sizes.

### 3.3. Pore pressure evolution during the seepage

**Figure 9.**
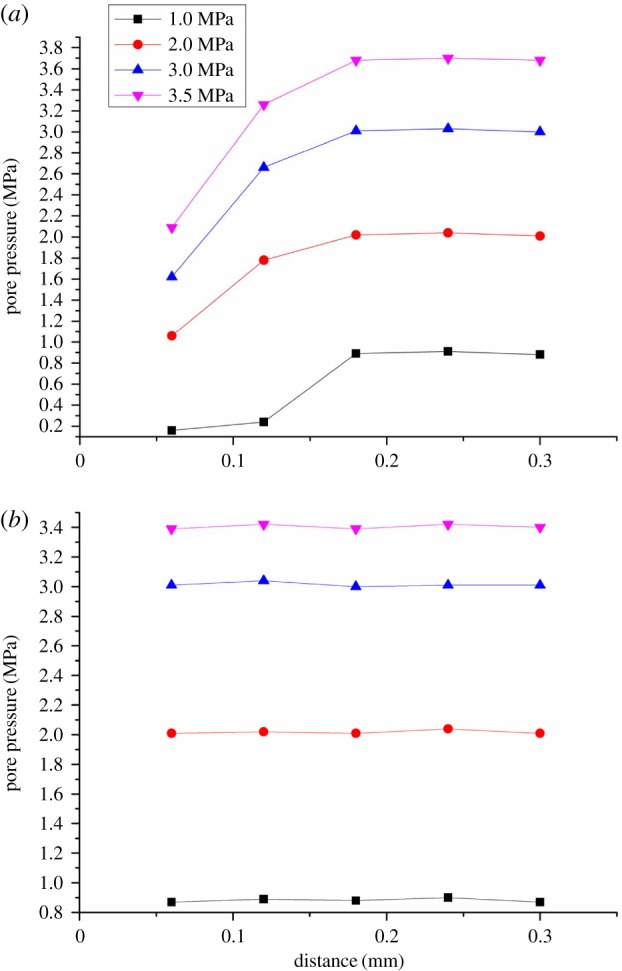
Distribution of pore pressure along the axial distance at 40 KN. (*a*) 2.5–5.0 mm, (b) 8.0–10.0 mm.

Along the tube, the distance of the pore 1, 2, 3, 4, 5 is a linear distribution (in figure 5). Figure 9*a,b* shows the pore pressure distribution of two particle sizes: 2.5–5.0 and 8.0–10.0 mm under the axial load of 40 kN. For 2.5–5.0 mm particle size, the change of pore pressure 1, 2 is not synchronized with the change of pore pressure 3, 4, 5. In figure 9*a*, the upper region of broken gangue becomes the plug which is the main part to block the flow of water.

It is mostly linear distribution for pore pressure 1, 2, 3, 4, 5. So the inner pore pressure of broken rock in the tube is almost linear, which is deduced from the pore pressure distribution of two particle sizes: 2.5–5.0 mm and 8.0–10.0 mm.

### 3.4. Mass loss during the seepage

The compacted gangue is dried, and screen mesh is used to screen the particle size after the test. The results are in table 4. The initial weight of broken gangue is 4.5 kg, different particle sizes of gangue have the different mass loss. For the gangue of 2.5–5.0, 5.0–8.0, 8.0–10.0, 10.0–12.0, 12.0–15.0 mm, the quality loss is 0.28, 0.25, 0.27, 0.14, 0.12 kg, respectively.

**Table 4.** Oven-dried particle size weight after seepage test.

	Oven-dried particle size weight after seepage test						
original particle type (mm)	0.0–2.5 mm (kg)	2.5–5.0 mm (kg)	5.0–8.0 mm (kg)	8.0–10.0 mm (kg)	10.0–12.0 mm (kg)	12.0–15.0 mm (kg)	mass loss (kg)
2.5–5.0	2.42	1.8					0.28
5.0–8.0	1.72	1.37	1.16				0.25
8.0–10.0	1.64	0.96	1.06	0.57			0.27
10.0–12.0	1.37	0.86	0.80	0.64	0.69		0.14
12.0–15.0	1.26	0.81	0.76	0.40	0.37	0.78	0.12

**Figure 10.**
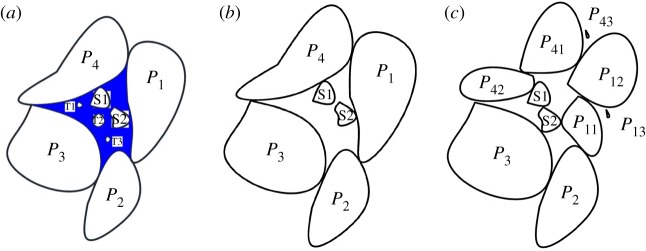
Course of quality loss: (*a*) initial phase, (*b*) seepage phase, (*c*) loading phase.

**Figure 11.**
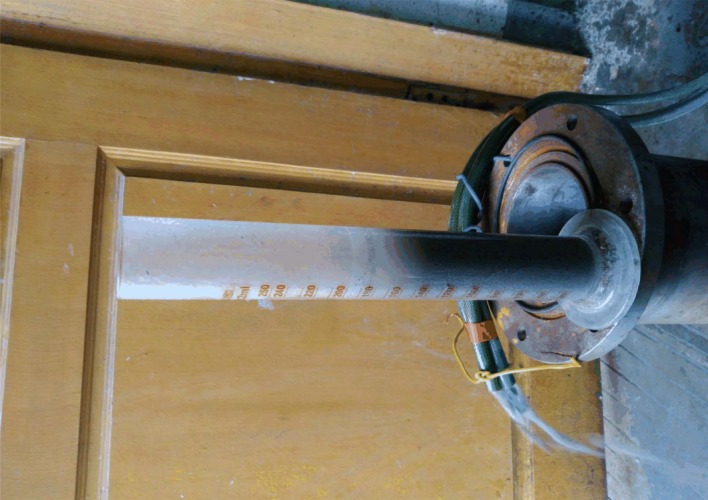
Water flow during the seepage (under axial pressure 40 kN).

Figure 10 shows the different phases of seepage, figure 10*a* shows the initial phase, where *P*_1_, *P*_2_ *P*_3_ and *P*_4_ are the big broken gangue, S1 and S2 are the small broken gangue, and T1 and T2 are tiny broken gangue. During the pore water flow, T1 and T2 are being carried off in figure 10*b*. Figure 10*c* shows big broken gangue are being further broken with big pressure, *P*_1_ changes into *P*_11_, *P*_12_ *P*_13_, and *P*_14_ changes into *P*_41_, *P*_42_ and *P*_43_. In figure 11, the cloudy mixture comes out of the seepage test device, which includes water and a lot of small particles. During the compaction, the broken gangue undergoes further fragmentation and restructuring; large particles break into small pieces and small particles. New superiority seepage path forms, which leads to the instability of seepage. The mass loss of cemented broken rocks is studied, in which brokenness of large particles needs to be studied further [21].

## 4. Conclusion

The results show that:

(1) Permeability parameter *k* decreases along with effective stress *σ*′*,* and non-Darcy factor *β* increases with effective stress *σ*′*.* The particle size of gangue has great influence on the permeability parameter *k* under effective stress 3 MPa. Non-Darcy factor *β* of small particle sizes increase quickly when the effective stress exceeds 2.0 MPa.
(2) The height of broken gangue does not show an increase in proportion to the particle size of broken gangue. The particle size of 8.0–10.0 mm has a good support effect.
(3) The inner pore pressure of broken rock in the tube is almost linear distribution.
(4) Different particle sizes of 4.5 kg broken gangue after seepage have the mass loss, and the weight is 0.28, 0.25, 0.27, 0.14 , 0.12 kg, respectively.

## Data accessibility

All original data are deposited at the Dryad Digital Repository: http://dx.doi.org/10.5061/dryad.r26t61c [32].
